# Electrocardiography in Hypertensive Patients without Cardiovascular Events: A Valuable Predictor Tool?

**DOI:** 10.1155/2022/7038894

**Published:** 2022-07-21

**Authors:** J. Ricardo Pires, M. Teixeira, F. Ferreira, I. Viseu, V. Afreixo, C. Neves

**Affiliations:** ^1^Internal Medicine Department, Centro Hospitalar Baixo Vouga, Aveiro, Portugal; ^2^University of Aveiro, Aveiro, Portugal

## Abstract

**Background:**

Hypertension is an important risk factor of cardiovascular (CV) disease. An early diagnosis of target organ damage could prevent major CV events. Electrocardiography (ECG) is a valuable clinical technique, with wide availability and high specificity, used in evaluation of hypertensive patients. However, the use of ECG as a predictor tool is controversial given its low sensitivity. This study aims to characterise ECG features in a hypertensive population and identify ECG abnormalities that could predict CV events.

**Methods:**

We studied 175 hypertensive patients without previous CV events during a follow-up mean of 4.0 ± 2.20 years. ECGs and pulse wave velocity were performed in all patients. Clinical characteristics and ECG abnormalities were evaluated and compared between the patients as they presented CV events.

**Results:**

Considering the 175 patients (53.14% male), the median age was 62 years. Median systolic blood pressure was 140  mmHg and diastolic blood pressure was 78 mmHg. Median PWV was 9.8 m/s. Of the patients, 39.4% were diabetic, 78.3% had hyperlipidaemia, and 16.0% had smoking habits. ECG identified left ventricular (LV) hypertrophy in 29.71% of the patients, and a LV strain pattern was present in 9.7% of the patients. Twenty-nine patients (16.57%) had a CV event. Comparative analyses showed statistical significance for the presence of a LV strain pattern in patients with CV events (*p*=0.01). Univariate and multivariate analysis confirmed that a LV strain pattern was an independent predictor of CV event (HR 2.66, 95% IC 1.01–7.00). In the survival analysis, the Kaplan–Meier curve showed a worse prognosis for CV events in patients with a LV strain pattern (*p*=0.014).

**Conclusion:**

ECG is a useful daily method to identify end-organ damage in hypertensive patients. In our study, we also observed that it may be a valuable tool for the prediction of CV events.

## 1. Introduction

Hypertension is a major risk factor for cardiovascular (CV) events. European arterial hypertension guidelines recommend a 12-lead electrocardiogram (ECG) as part of the routine evaluation of all hypertensive patients to assess organ damage [1]. In fact, ECG is a widely available non-invasive and relatively inexpensive technique, thus making it easy to execute and reproducible [2, 3]. In hypertensive patients, it is possible to detect many abnormalities on a resting ECG mirroring cardiac pathologic changes [2, 4].

The most described signs are related to left ventricular hypertrophy, but atrial deformities are also noticeable [5–11].

There has been a high demand and need to find high cost-effective methods to predict CV events. Despite many studies having identified the values of ECG as a predictor of events in hypertensive patients, the use of ECG as a predictor tool remains controversial due to its low sensitivity [12–14].

With this study, the authors propose to characterise ECG features in hypertensive patients and identify ECG abnormalities that could predict CV events.

## 2. Methods

The data source was a cohort of treated hypertension patients in a hypertension outpatient clinic that was followed, without previous CV events. A 12-lead surface ECG was recorded for all patients in the supine position using ELI™ 280 electrocardiograph with a paper speed of 25 mm/s and 10 mm/mV standardisation. ECGs were transferred to digital media, and trained readers performed the measurements. The ECG data recorded are explained in supplement 1.

Additionally, a pulse wave velocity (PWV) was performed using the Complior device (Alam Medical, France) and the clinical information of vascular risk factors and biometric data was collected.

Patients with previous CV events, secondary hypertension, pregnancy, and ECG with atrial fibrillation/atrial flutter or left/right bundle branch block were excluded.

The patients' hospital physician diagnosed the CV event, or it was found in their medical records. CV events included cerebrovascular disease, coronary heart disease, heart failure with hospitalisation, and surgery for peripheral artery disease.

All statistical analyses were performed using R version 4.0.2. Baseline characteristics were reported as percentages (%) for categorical variables and medians with IQR for continuous variables. Baseline and ECG characteristics were compared between patients who developed a CV event and patients without CV events. Characteristics were compared using the Mann–Whitney *U* test for non-normally distributed continuous data and the chi-square test for categorical variables. Univariate and multivariate Cox and proportional hazards analyses were performed to identify ECG abnormalities significantly associated with a future CV event. The primary event-free rates between the two groups were estimated using Kaplan–Meier analyses. A *p* value of less than 0.05 was considered statistically significant.

## 3. Results

A total of 175 patients were included in this study. Patients' baseline characteristics are given in Table 1. The median age of patients was 62 (IRQ 13) years, and 94 patients (53.14%) were male. The median BMI was 28.67 kg/m^2^, the median heart rate was 69 beats per minute, the median systolic and diastolic pressures were 140 mmHg and 78 mmHg, respectively, and the median PWV was 9.8 m/s. Regarding risk factors of all of the patients, 39.40% were diabetic, 78.30% had dyslipidemia, and 16.00% were smokers.

Of all of included patients, with a follow-up mean of 4.0 ± 2.20 years, 29 had a CV event (17 cerebrovascular events, 9 coronary events, 2 heart failures with hospitalisation, and 1 surgery for peripheral artery disease). Baseline characteristics were homogeneous between the groups; however, patients with a CV event were more likely to be male with a higher prevalence of CV risk factors, such as diabetes and dyslipidemia (Table 1).

**Figure 1:**
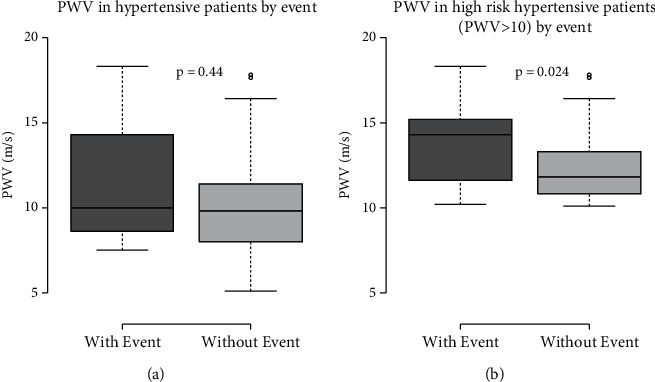
Pulse wave velocity (PWV) in hypertensive patients.

There was no significant difference in aortic stiffness, expressed as aortic PWV, between the groups. However, when analysing only very high-risk patients (PWV values > 10 m/s), the group with CV had much higher PWV values than patients without CV (*p*=0.024) (Figure 1).

Regarding ECG characteristics (Table 2), overall, patients had a median QRS duration of 101 ms (IQR 19), with a correct QT interval of 421 ms (IQR 34.5).

**Figure 2:**
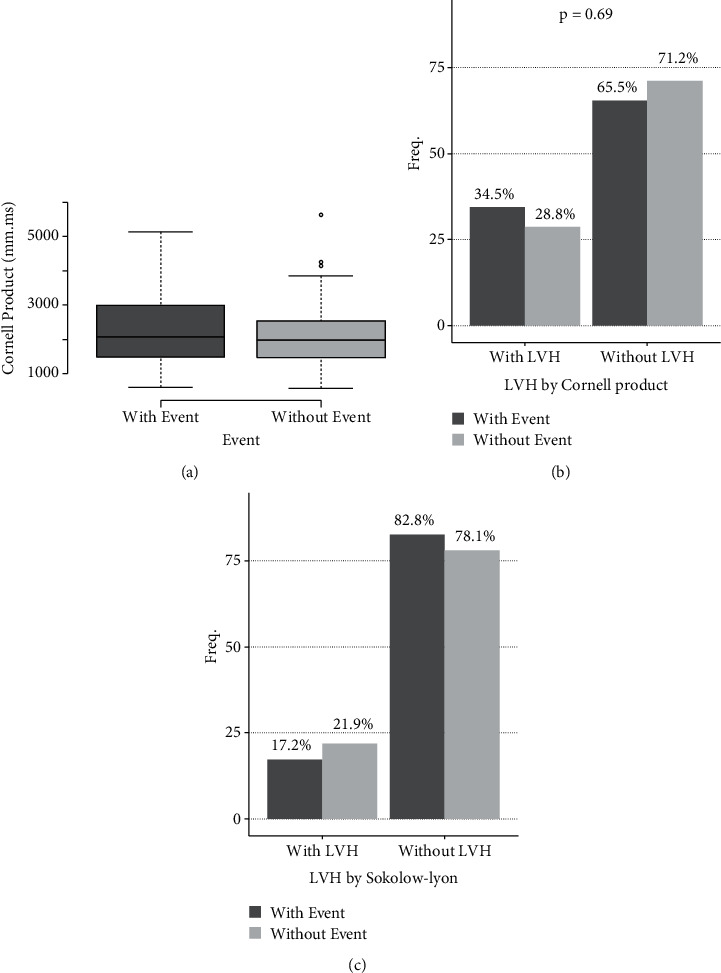
Cornell and Sokolow–Lyon criteria.

**Figure 3:**
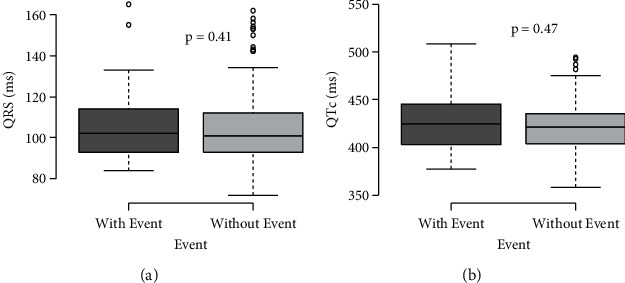
QRS duration and QT interval in hypertension patients.

Left ventricular hypertrophy was identified in 21.10% of patients using the Sokolow–Lyon criteria and 29.71% using the Cornell criteria (Figure 2). In both groups, QRS values and the correct QT interval were similar (Figure 3). The prevalence of left ventricular hypertrophy was higher in the group with CV events.

**Figure 4:**
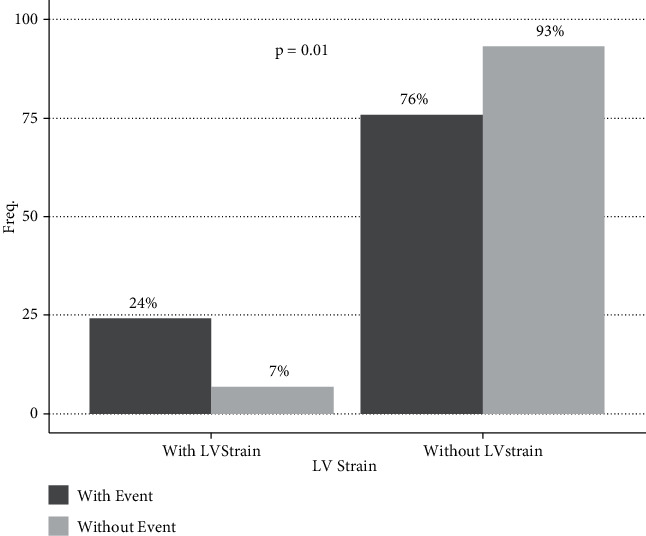
Left ventricular Strain pattern.

Patients in this group also presented with a significantly more frequent strain pattern on ECG than patients without CV events (Figure 4).

**Figure 5:**
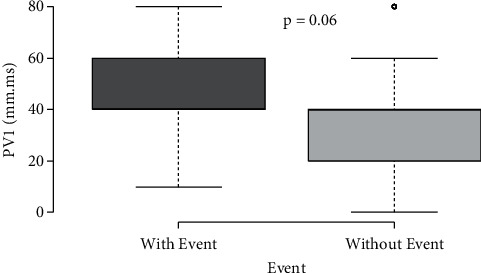
P-wave terminal force in V1.

Despite the median value of P-wave terminal force being similar between the groups, the group with CV had higher values (Figure 5).

**Figure 6:**
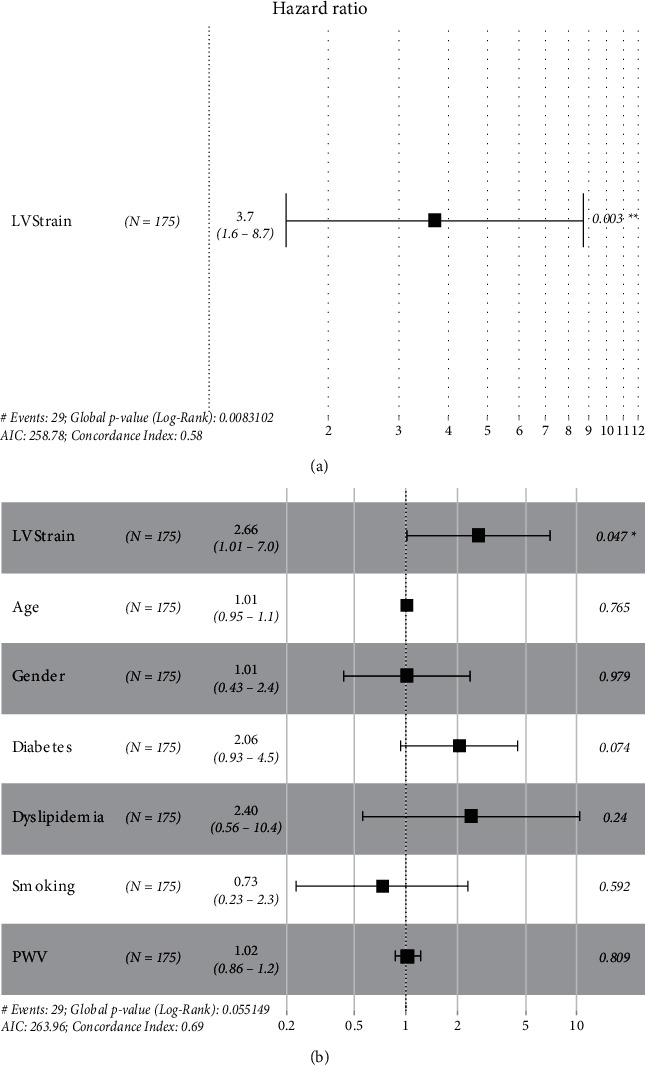
Hazard ratio.

**Figure 7:**
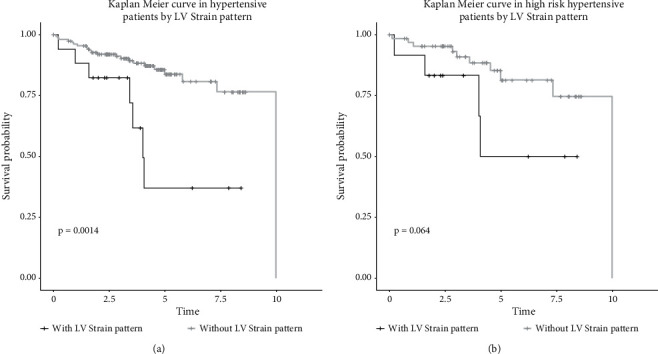
Kaplan–Meier curve in hypertensive patients.

Univariate Cox (Figure 6) proportional hazards regression analyses showed that left ventricular strain pattern was significantly associated with CV events (HR 3.7, 95% IC 1.6–8.7). A multivariate analysis adjusted for clinical characteristics confirmed that the left ventricular strain pattern was an independent predictor of the event (HR 2.66, 95% IC 1.01–7.00). Furthermore, a Kaplan–Meier (Figure 7) analysis showed a significantly lower primary event-free rate in patients without a left ventricular strain pattern (*p* < 0.05), and the same tendency was shown when only high-risk patients were selected (PWV >10 m/s).

## 4. Discussion

Hypertension leads to structural vascular and cardiac changes, reflected in ECG abnormalities [2]. In addition, hypertensive patients, despite reasonable control, such as in our study, have a higher risk of developing CV events. It is important to identify easy tools to alert clinicians of patients who are more at risk. PWV is a useful and widely recognized tool to predict vascular events [15]. Although our sample patients who developed CV events had the same median value of PWV, when we only selected high-risk patients, we observed higher values in the group who presented CV events. Thus, PWV could be a good examination for high-risk patients, even in the patients with resistant hypertension.

ECG is a versatile technique that is easier to perform than PWV and allows for the evaluation of organ damage in hypertensive patients over time. ECG abnormalities increase with the increasing severity in hypertension [3]; however, minor changes could be predictors of CV events [4]. In our study, we selected the most described ECG features related to hypertension [2, 3, 14].

Left ventricular hypertrophy is the hallmark of the effect of hypertension on the heart and can be easily assessed used ECG according to various criteria, with the Cornell and Sokolow–Lyon criteria being the most used [5, 7, 8, 16]. In our sample, as well as in the many other studies, the Cornell criteria were able to identify more individuals with left ventricular hypertrophy compared to the Sokolow–Lyon criteria [16]. The prevalence of left ventricular hypertrophy was 29.71% higher than in reviewed studies [2, 17, 18]. Conversely, no significant differences were observed between patients with CV and those without (Figure 2).

QRS duration corresponds to ventricular depolarization, and prolonged QRS intervals may reflect myocardial hypertrophy [19]. It is described as a prolonged QRS duration in hypertensive patients, but its prognostic value is not well established. In our sample, half of the patients had a QRS interval that extended beyond 100 ms; however, no differences were observed between patients with CV events and those without (Figure 3).

A prolonged corrected QT interval is defined as at least 450 ms in men and 460 ms in women [20]. A correct QT interval prolongation has been described in hypertensive patients, but it was not observed in our sample. This could be related to antihypertensive treatment since these drugs have a beneficial effect in reducing QT interval, especially those that inhibit the renin-angiotensin-aldosterone system [20](Figure 3).

Another well-recognized marker for the presence of a higher left ventricular mass, lower myocardial contractility, and myocardial fibrosis is the left ventricular strain pattern [6, 8]. Similar to other studies, in our sample, the prevalence of this strain pattern in patients who develop a CV event was significantly higher (24%) compared to patients without CV events (Figure 4).

The P-wave terminal force in V1 is an emerging factor as a strong predictor of CV events [10, 11]. It is related to the enlargement of the left atrium, and abnormal values were defined as ≥40 mm × ms. In our sample, patients with CV tend to have a P-wave terminal force of <40 mm × ms; however, this feature is not a predictor of CV event (Figure 5).

Given the differences between the groups, the hazard ratio (Figure 6) of the left ventricular strain pattern was calculated using the Cox model, showing that the left ventricular strain pattern was significantly associated with CV events in the univariate model (HR 3.7, 95% IC 1.6–8.7) and in multivariate analysis adjusted for clinical characteristics, such as other CV risk factors and PWV (HR 2.66, 95% IC 1.01–7.00). In the survival analysis, the Kaplan–Meier curve (Figure 7) showed a worse prognosis for CV events in patients with a left ventricular strain pattern (*p*=0.014), even when only the patients with higher CV risk were selected (PWV >10 m/s).

## 5. Conclusion

In conclusion, this study contributes to the growing understanding of changes in the ECG associated with hypertension and added value of this tool in the prediction of CV events. The ECG strain pattern is a marker of left ventricular hypertrophy. It seems to be an independent CV event predictor in hypertensive patients, and clinicians should be alerted to the existence of this pattern.

## Figures and Tables

**Table 1:** Baseline characteristics.

	Global population		Without CV event		With CV event		*p* value
	Median (IQR)	Freq. (%)	Median (IQR)	Freq. (%)	Median (IQR)	Freq. (%)	
Age (years)	62 (13)	—	62 (13)	—	64 (12)	—	0.44
Gender (male)	—	53.1	—	51.4	—	62.1	0.39
BMI (Kg/m^2^)	28.7 (6.1)	—	28.9 (6.1)	—	30.8 (4.9)	—	0.22
HR (bpm)	69 (16)	—	69.5 (16.5)	—	69 (15.0)	—	0.75
SBP (mmHg)	140 (22)	—	139 (23.5)	—	143 (21.5)	—	0.11
DBP (mmHg)	78 (16)	—	78 (15)	—	78 (19)	—	0.56
PWV (m/s)	9.8 (3.6)	—	9.8 (3.4)	—	10 (5.7)	—	0.09
Diabetes	—	39.4	—	34.9	—	62.1	0.01
Dyslipidemia	—	78.3	—	76.0		89.7	0.17
Smoking	—	16.0	—	16.4	—	13.8	1

**Table 2:** ECG characteristics.

	Global population		Without CV event		With CV event		*p* value
	Median (IQR)	Freq. (%)	Median (IQR)	Freq. (%)	Median (IQR)	Freq. (%)	
QRS (ms)	101 (19)	—	101 (18.5)	—	102 (21)	—	0.41
QTc (ms)	421 (34.5)	—	421 (30.8)	—	425 (42)	—	0.47
Cornell product (mm.ms)	1995 (1119.5)	—	1986 (1063)	—	2071 (1508)	—	0.47
PV1 (mm.ms)	40 (20)	—	40 (20)	—	40 (20)	—	0.06
LV strain	—	9.7	—	24.1	—	6.8	0.01
LVH (Sokolow–Lyon criteria)	—	21.1	—	21.9	—	17.2	0.75

## Data Availability

The datasets used and/or analysed during the current study are available from the corresponding author on reasonable request.

## Supplementary Materials

Supplementary Materials Supplementary Data: overview of ECG parameters used in this study.
