# Supplementary material for: Electrocardiography in Hypertensive Patients without Cardiovascular Events: A Valuable Predictor Tool?
Source: Int J Hypertens. 2022 Jul 21;2022:7038894. doi: 10.1155/2022/7038894 (PMC9334108; doi:10.1155/2022/7038894)
Supplement: Supplementary Materials — Supplementary Data: overview of ECG parameters used in this study. [file 7038894.f1.docx]

**Supplementary Data**

Overview of ECG parameters

*Supplement 1*

| P wave terminal force | Product of the duration and amplitude of the negative terminal deflection on the P wave in V1 |
| --- | --- |
| QTc | QT interval corrected for heat rate |
| QRS duration | Average of the first deflection from the isoelectric line after the P wave until the J-point |
| LVH by Sokolow–Lyon criteria | S in V1 + R in V5 or V6 ≥35 mm, or R in aVL ≥ 11mm |
| LVH Cornell criteria | SV3 RaVL >2.8mV (men) >2.0mV (women) |
| Cornell voltage product | (RaVL+SV3) x QRS duration (msec) ≥ 2440 |
| LV strain pattern | ST depression and T-wave inversions in I, aVL and V5-6 |
